# Diffuse midline glioma with H3-K27M mutation: A rare case with GFAP-positive anucleate whorled patterns

**DOI:** 10.1097/MD.0000000000029448

**Published:** 2022-06-17

**Authors:** Yi-Hua Wang, Jian Gu, Juan-Han Yu, Lin Fu, Qing-Chang Li, Xue-Shan Qiu, En-Hua Wang

**Affiliations:** Department of Pathology, The First Affiliated Hospital of China Medical University, Shenyang, P.R. China.

**Keywords:** diffuse midline gliomas, glial fibrillary acidic protein-positive anucleate whorled patterns, H3-K27M mutant, molecular genetic analysis, third ventricle

## Abstract

**Introduction:**

Diffuse midline glioma with H3-K27M mutation is an infiltrative high-grade glioma, with predominantly astrocytic differentiation.

**Patient concerns:**

A 54-year-old Chinese woman presented with memory loss for a month and walking instability for 15 days.

**Diagnosis:**

Magnetic resonance imaging showed a mass shadow of isometric T1 and slightly longer T2 with mild mixed signals in the third ventricle of the suprasellar region. Histologically, the tumor was primarily sheet-like, with many “anucleate areas” composed of long and thin fibrillary processes of the bipolar cells, which formed “whorls.” The neoplastic nuclei were ovoid and moderate in size. The tumor showed brisk mitotic activity and vascular proliferation, with no necrosis. In addition to histone H3K27M mutation, immunohistochemical staining showed that the tumor cells were positive for glial fibrillary acidic protein, oligodendrocyte transcription factor 2, alpha-thalassemia/mental retardation syndrome X, S-100 and Vimentin. The “anucleate areas” were positive for glial fibrillary acidic protein and negative for synaptophysin. The Ki-67 proliferation index was about 10%. Molecular genetic analyses detected H3F3A K27M mutation, but no mutations in IDH1 or IDH2, TERT promoter mutations, MGMT promoter methylation, KIAA1549-BRAF fusion or deletion of 1p/19q were found. Based on these findings, the patient was diagnosed as diffuse midline glioma with H3-K27M mutation in the third ventricle, corresponding to WHO grade 4.

**Interventions:**

A craniotomy with total excision of the tumor was performed.

**Outcomes:**

After surgery, she was routinely treated with temozolomide for chemotherapy and synchronous radiotherapy. It has been 11 months now, and the patient is living well.

**Conclusion:**

This case report provides information on the microscopic morphological features of diffuse midline glioma with H3K27M mutation, which can help pathologists to make a definitive diagnosis of this tumor.

## 1 Introduction

Diffuse midline glioma with H3-K27M mutation is a new tumor type in the 2016 WHO classification of tumors of the central nervous system (CNS),^[1,2]^ with predominantly astrocytic differentiation and is characterized by K27M mutation in either H3F3A/-HIST1H3B/C. It occurs at the midline (spinal cord, thalamus, brainstem, cerebellum, hypothalamus, third ventricle, pineal gland, etc.)^[3–7]^ of the CNS. It is mainly found in children, and adult cases are relatively rare.^[3,8]^

Although its histopathological features have been widely described, glial fibrillary acidic protein (GFAP)-positive anucleate whorled patterns have not been reported to the best of our knowledge. Hence, we have reported this unusual case.

## 2 Clinical summary

**Figure 1:**
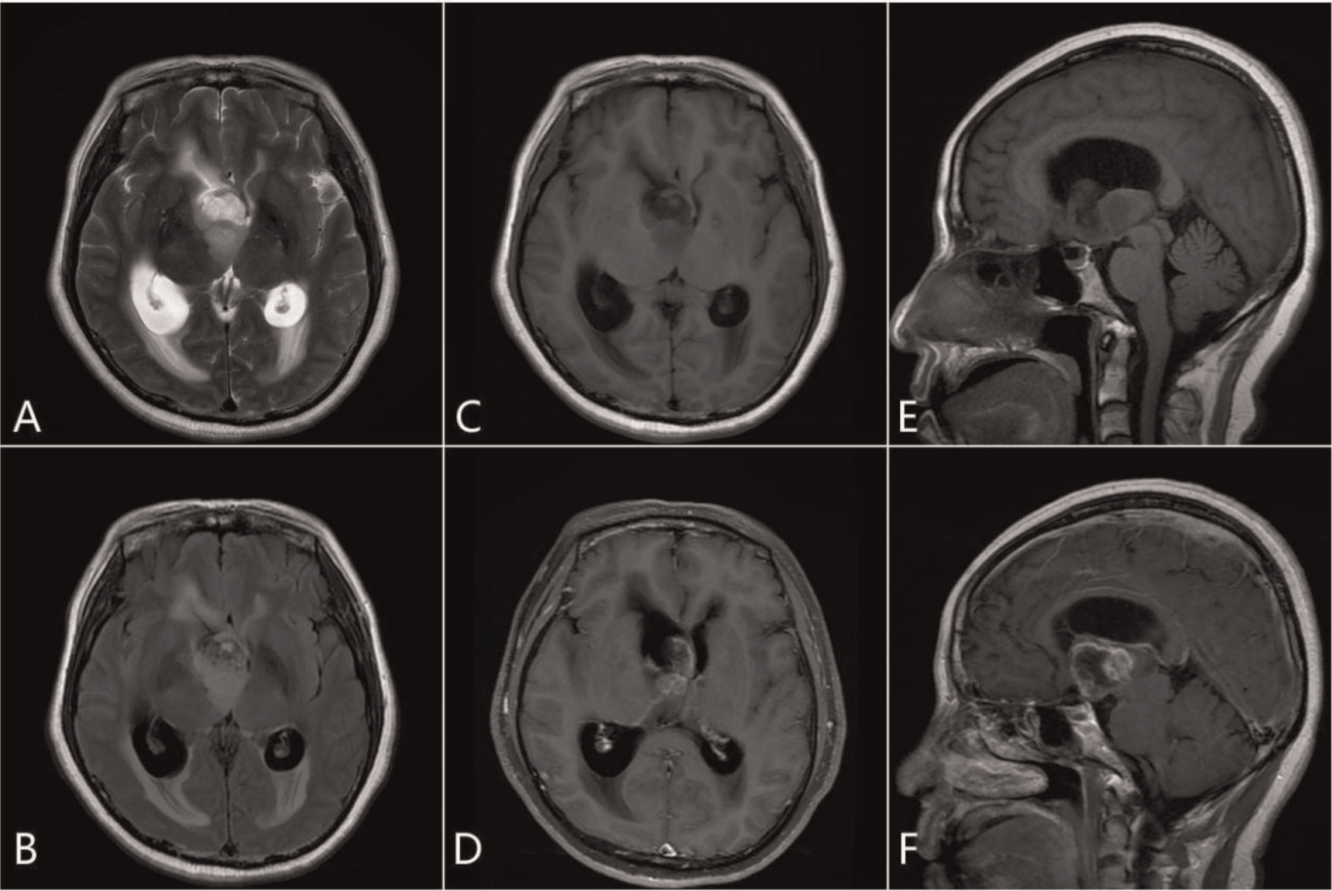
The third ventricle showed isometric T1, slightly longer T2 with mild mixed signal mass shadow, cystic solid. The boundary was clear, with no definite edema signal in the adjacent brain tissue. The main body of the lesion was located in the third ventricle and spread to the right lateral ventricle. Bilateral ventricular dilatation was observed, wherein the right ventricle was more significant. A long stripe T2 signal can be seen at the edge of the lateral ventricle (interstitial brain edema). Enhanced scanning of the mass sac wall and solid components showed irregular ring enhancement, and local lace-like enhancement (A, axial T2; B, axial T2 FLAIR; C, axial T1; D, axial T1 enhancement; E, sagittal T1; F, enhanced sagittal T1).

A 54-year-old Chinese female developed memory loss for half a month, which was characterized by intermittent short-term memory loss, but her long-term memory remained normal. After 15 days, the patient's memory loss obviously worsened, and both long-term memory and short-term memory were poor. Concurrently, she also developed walking instability. Magnetic resonance imaging showed a mass shadow of isometric T1 and slightly longer T2 with mild mixed signals in the third ventricle of the suprasellar region, about 3.4 × 2.5 × 3.4 cm in size. The enhanced scan showed that the lesion was irregular and annular, with a clear edge. The lesion had spread to the right lateral ventricle, which dilated the ventricular system, especially the right lateral ventricle. Furthermore, the lamellar long T2 signal was seen in bilateral paraventricular white matter, the midline structure had shifted to the left, and the corpus callosum was thinner (Fig. 1). Radiological findings suggested ependymoma. Subtotal resection craniotomy was performed. After surgery, the patient was routinely treated with temozolomide for chemotherapy and synchronous radiotherapy (Dt = 60 Gy/30f). It has been 11 months now, and the patient is living well.

## 3 Histopathological and immunohistochemical methods

The resected specimens were fixed with 10% neutral buffer formaldehyde and embedded with paraffin. Thereafter, the tissue was cut into 4 μm sections, decarbonized in xylene, rehydrated with graded alcohols, and immunostained with the following antibodies: alpha-thalassemia/mental retardation syndrome X, GFAP, isocitrate dehydrogenase (IDH), neuronal nuclear antigen (NeuN), oligodendrocyte transcription factor 2, S-100, synaptophysin (Syn), Vimentin and Ki-67 (MaiXin, China). All samples were midly counterstained with hematoxylin, dehydrated and mounted. The negative controls were incubated with PBS instead of the primary antibody, using the same procedure.

Fluorescent in situ hybridization was performed to check for deletions of chromosomes 1p and 19q. Dual color-probe hybridization was performed with Vysis 1p36/1q25 and 19q13/19p13 fluorescent in situ hybridization probe kit (Abbott Molecular, Des Plaines, IL, Illinois) according to the manufacturer's instructions. At least 100 non-overlapping nuclei were counted; samples were considered to be 1p- or 19q-deleted when >30% of counted nuclei presented one target (red) signal and two reference (green) signals. Sanger sequencing was used to detect the IDH 1/2 mutation and TERT promoter mutations. K27M mutation and KIAA1549-BRAF fusion were performed by Sinomdgene company (Beijing, China) and Kanglu company (Hubei, China), respectively.

## 4 Pathological and genetic findings

**Figure 2:**
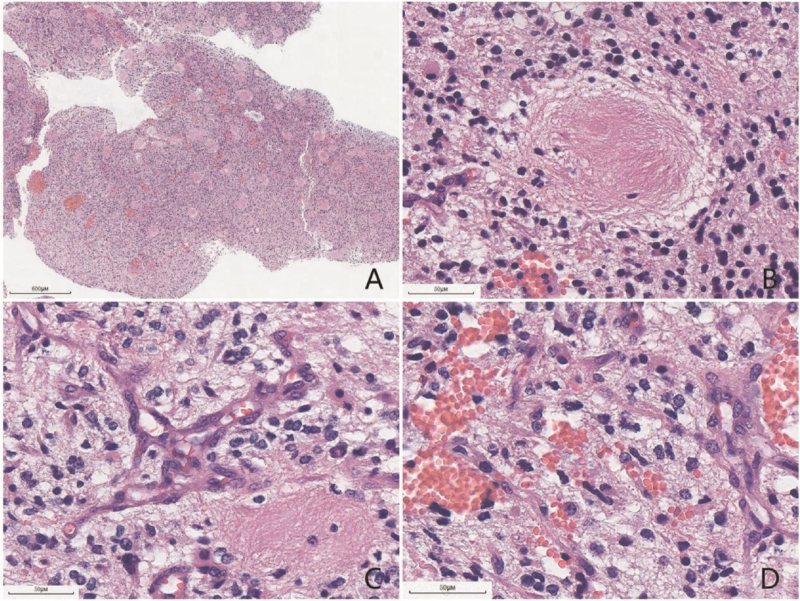
The resected tumor was primarily sheet-like (scale bar: 600 μm) (A), and the spindle-shaped tumor cells with thin, elongated processes formed “whorls” (scale bar: 50 μm) (B). Microvascular proliferation (scale bar: 50 μm) (C) and brisk mitotic activity (scale bar: 50 μm) (D) were observed.

**Figure 3:**
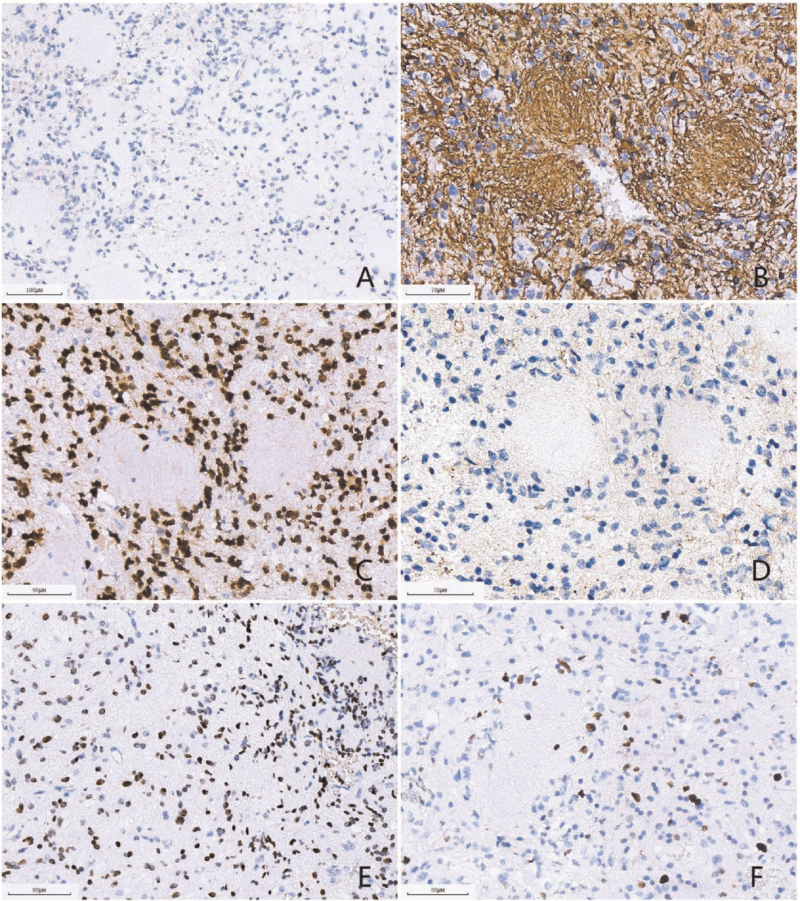
(A) (scale bar: 100 μm) The tumor cells were negative for IDH1 R132H. (B) (scale bar: 70 μm) The tumor cells and “neuropil-like islands” were positive for GFAP. (C) (scale bar: 90 μm) The tumor cells were positive for Olig-2. (D) (scale bar: 70 μm) The tumor cells and “neuropil-like islands” were negative for synaptophysin. (E) (scale bar: 90 μm) The tumor cells were positive for H3-K27 M mutant protein. (F) (scale bar: 80 μm) The Ki-67 proliferation index was about 20%.

**Figure 4:**
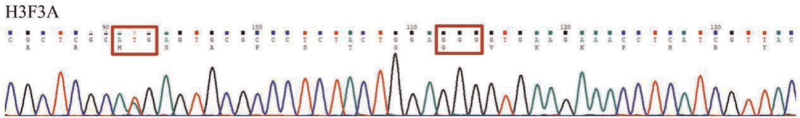
Sequence analysis showed mutation at position K27M in the histone coding gene H3F3.

Small grayish-red fragments of the resected lesion were sent for histological examination. The lesion was 1.2 × 0.7 × 0.3 cm in size. Histological examination with hematoxylin and eosin staining revealed that the tumor was primarily sheet-like, and the tumor cells showed moderate cellularity. Notably, there were many “anucleate areas” in the tumor, composed of long and thin fibrillary processes of the bipolar cells, which formed “whorls.” The neoplastic nuclei were ovoid and moderate in size. Moreover, the tumor showed brisk mitotic activity and vascular proliferation, with no necrosis (Fig. 2). Immunohistochemical staining revealed that the tumor cells were positive for GFAP, oligodendrocyte transcription factor 2, histone H3K27M mutant protein, alpha-thalassemia/mental retardation syndrome X, S-100 and Vimentin, but negative for IDH1 R132H, Syn and NeuN. The “anucleate areas” were positive for GFAP, but negative for Syn. The Ki-67 proliferation index was about 10% (Fig. 3). Molecular genetic analyses detected *H3F3A* K27M mutation (Fig. 4), while no mutations in *IDH*1 or *IDH*2, *TERT* promoter mutations, *MGMT* promoter methylation, *KIAA1549-BRAF* fusion or deletion of 1p/19q were found. Based on these findings, the patient was diagnosed with diffuse midline glioma with H3-K27M mutation in the third ventricle, corresponding to WHO grade 4.

## 5 Discussion

According to the 2016 WHO classification of the CNS and cIMPACT Working Committee 3 for diagnosing diffuse midline glioma with H3-K27M mutation, a tumor should be diffuse, midline glioma, with H3 K27M mutation.^[1,2]^ The histological features of diffuse midline gliomas are diverse. The relatively rare morphological characteristics include epithelioid and rhabdoid cells, gliomas with giant cells, ependymal-like areas, primitive neuroectodermal tumor-like foci, sarcomatous transformation, ganglionic differentiation and pleomorphic xanthoastrocytoma-like areas, pilomyxoid features, and “rosette-like” nerve islands, neuropil-like islands.^[4,7]^ Notably, Gao et al reported a diffuse midline glioma with primitive neuroectodermal tumor-like and neuropil-like islands.^[4]^ The neuropil-like islands were similar to the anucleate whorled structures in the current case, and both of them appeared as vortex-like islands. The difference was that the neuropil-like islands showed neuronal differentiation in the previous study and were positive for Syn, with scattered NeuN-positive neuronal cells.^[4]^ In contrast, in our case, the nuclear-free zone was formed by the extension of slender cytoplasmic processes by GFAP-positive glial cells, but was negative for neuronal markers. Moreover, the tumor cells showed positive expression of H3K27M by immunohistochemistry, and gene detection further confirmed that they contained H3K27M gene mutation. Based on these findings, we diagnosed the tumor as diffuse midline glioma with histone H3-K27M mutation in the third ventricle. Given the varied histological features of diffuse midline gliomas, understanding the location and genetic alterations can help with the correct diagnosis.

Poor prognosis of most H3K27M mutant gliomas has been confirmed in many studies,^[3,4,9–11]^ and less than 10% of patients survive for more than 2 years,^[1]^ so this tumor is classified as WHO grade 4. Some researchers noted that H3K27M mutant gliomas may also show histological grade 2/3,^[3,9,11]^ and the median overall survival rate of H3K27M mutation cases with histological grade 2/3 is significantly longer than those with histological grade 4.^[9,11]^ Therefore, histological classification plays a very important role in prognosis. In this case, the density of the cells under the microscope was medium, and vascular proliferation was notable, but there was no obvious necrosis, and histology indicated WHO grade 3. The patient was routinely treated with temozolomide for chemotherapy and synchronous radiotherapy (Dt = 60 Gy/30f) after surgery. To date, she has not had a recurrence for 11 months. The association between prognosis and histological grade of diffuse midline glioma needs further validation.

Herein, we described an extremely rare case of diffuse midline glioma, H3-K27M mutation with GFAP-positive anucleate whorled patterns. This case report provides information on the microscopic morphological features of diffuse midline glioma with H3K27M mutation, which can help pathologists to make a definitive diagnosis of this tumor.

## Acknowledgments

The authors thank the technical assistance in genetic analysis from Beijing Sino MD gene Technology Co., Ltd and Wuhan Kanglu Medical Laboratory Co., Ltd

## Author contributions

**Investigation:** Qing-Chang Li.

**Methodology:** Jian Gu.

**Resources:** Lin Fu.

**Software:** Xue-Shan Qiu.

**Supervision:** En-Hua Wang.

**Writing – original draft:** Yi-Hua Wang.

**Writing – review & editing:** Juanhan Yu.
